# Understanding E2 versus S_N_2 Competition under Acidic and Basic Conditions

**DOI:** 10.1002/open.201300043

**Published:** 2014-01-29

**Authors:** Lando P Wolters, Yi Ren, F Matthias Bickelhaupt

**Affiliations:** [a] Department of Theoretical Chemistry, Amsterdam Center for Multiscale Modeling, VU University AmsterdamDe Boelelaan 1083, 1081 HV Amsterdam (The Netherlands); [b] College of Chemistry and Key State Laboratory of Biotherapy, Sichuan UniversityChengdu 610064 (P. R. China); [c] Institute for Molecules and Materials, Radboud University NijmegenHeyendaalseweg 135, 6525 AJ Nijmegen (The Netherlands)

**Keywords:** activation-strain analysis, density functional calculations, elimination reactions, nucleophilic substitutions, reaction mechanisms

## Abstract

Our purpose is to understand the mechanism through which pH affects the competition between base-induced elimination and substitution. To this end, we have quantum chemically investigated the competition between elimination and substitution pathways in H_2_O+C_2_H_5_OH_2_^+^ and OH^−^+C_2_H_5_OH, that is, two related model systems that represent, in a generic manner, the same reaction under acidic and basic conditions, respectively. We find that substitution is favored in the acidic case while elimination prevails under basic conditions. Activation-strain analyses of the reaction profiles reveal that the switch in preferred reactivity from substitution to elimination, if one goes from acidic to basic catalysis, is related to (1) the higher basicity of the deprotonated base, and (2) the change in character of the substrates LUMO from C^β^−H *bonding* in C_2_H_5_OH_2_^+^ to C^β^−H *antibonding* in C_2_H_5_OH.

## Introduction

**Scheme 1:**
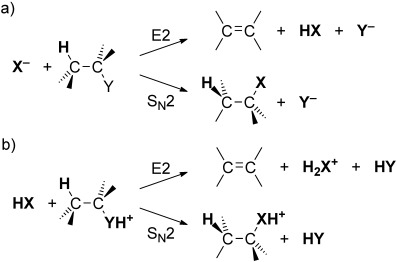
Model E2 and S_N_2 reactions corresponding to a) basic and b) acidic conditions.

Bimolecular base-induced 1,2-elimination (E2) is one of the most elementary reactions in organic chemistry.[1–6] Typically, in E2 reactions, an anionic base abstracts a proton from the β-carbon center of a substrate molecule while, simultaneously, a leaving group at the α position is released, as shown in Scheme 1 a. Elimination reactions are, in principle, in direct competition with another textbook organic reaction, namely, bimolecular nucleophilic substitution (S_N_2).[1] In the case of the reaction shown in Scheme 1 a, the competing S_N_2 reaction results from the anionic reactant carrying out a nucleophile attack at the α-carbon center, leading to release of the same leaving group as in the E2 reaction. E2 elimination has been observed to be favored by stronger bases. In particular, acid catalysis, which involves a comparatively weak, neutral base or nucleophile, generally goes with substitution, whereas basic conditions, often featuring stronger, anionic bases, tune reactivity towards elimination.

Elimination and substitution reactions have been studied also extensively in the gas phase using mass spectrometry techniques.[7–15] Note that differentiation between E2 and S_N_2 processes of the anionic reaction systems through mass spectrometry is problematic, requiring additional techniques or a special design of reactants (e.g., leaving group connected to substrate via a second bond) as both pathways lead, in principle, to the same detectable anionic product, namely, the leaving group Y^−^ (see Scheme 1 a). This problem does not occur when studying the corresponding reactions after protonation of nucleophile/base and leaving group (Scheme 1 b).[16, 17, 18] The resulting cationic reaction systems lead to different product ions for the respective mechanistic pathways, namely the conjugate acid XH_2_^+^ for E2 and the substituted substrate for S_N_2, which in general have a different *m*/*z* ratio that allows for straightforward characterization of the active mechanisms.

Theoretical studies on both anionic and cationic E2 and S_N_2 reactions have provided detailed information on reaction potential energy surfaces (PES) and the structure of species and transition states (TS).[19–27] Activation-strain analyses[28] of the anionic substitution reactions between halides and halomethanes[29] have shown that the S_N_2 barrier decreases as the highest-occupied molecular orbital (HOMO) of the nucleophile becomes a better electron donor, that is, as the basicity of the nucleophile becomes stronger, because of a more stabilizing interaction between nucleophile and substrate. Likewise, a weaker bond between carbon and the leaving group (C−LG) also leads to a lower S_N_2 barrier because the energetic strain associated with such a weaker bond is less destabilizing.[28, 29]

Herein, we wish to gain insight into the electronic mechanism behind the observed shift from substitution to elimination if one goes from acidic to more basic conditions. To this end, we have analyzed the competition between antiperiplanar 1,2-elimination and backside substitution in the cationic and anionic model systems H_2_O+CH_3_CH_2_OH_2_^+^ [Equations (1)] and OH^−^+CH_3_CH_2_OH [Eqs. (2)], using density functional theory (DFT). Our model systems represent, in a generic manner, one particular reaction under acidic and basic conditions, respectively.


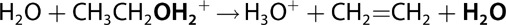
(1a)


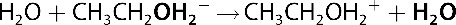
(1b)


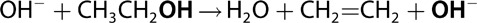
(2a)


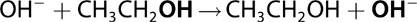
(2b)

Our computations show that indeed substitution is favored in the acidic case whereas elimination prevails under basic conditions. Activation-strain analyses of the reaction profiles reveal that the switch in preferred reactivity from substitution to elimination, if one goes from acidic to basic catalysis, is directly related to the significantly higher proton affinity of the anionic base. Deprotonation enhances the attack of the base in both pathways, elimination and substitution, but this effect is counteracted by a stronger C−LG bond in the neutral substrate. Interestingly, however, our activation-strain analyses show that protophilic attack benefits more from increasing the basicity than nucleophilic attack due to a different composition of the substrate LUMO under different reaction conditions.

## Theoretical Methods

### Computational details

All calculations were carried out using the Amsterdam Density Functional (ADF) program[30] and the Quantum-regions Interconnected by Local Descriptions (QUILD) program.[31] The numerical integration was performed using the procedure developed by te Velde et al.[32] The molecular orbitals (MOs) were expanded in a large uncontracted set of Slater-type orbitals (STOs): TZ2P (no Gaussian functions are involved). The TZ2P basis set[33] is of triple-*ζ* quality for all atoms and has been augmented with two sets of polarization functions, that is, 2p and 3d on H, and 3d and 4f on C and O. An auxiliary set of s, p, d, f and g STOs was used to fit the molecular density and to represent the Coulomb and exchange potentials accurately in each self-consistent field (SCF) cycle. All electrons are included in the variational treatment (no frozen-core approximation used).

Energies and geometries were calculated at the OLYP level of the generalized gradient approximation (GGA), which involves the optimized exchange (OPTX) functional proposed by Handy and coworkers,[34] and the gradient-corrected functional of Lee, Yang and Parr for correlation.[35] Scalar relativistic effects were accounted for using the zeroth-order regular approximation (ZORA).[36] Previous ab initio benchmark studies show that the OLYP functional, combined with the TZ2P basis set, leads to the same trends and qualitative features of the potential energy surfaces as CCSD(T)/aug-cc-pv(T+d)Z.[37]

Equilibrium structures were obtained by optimizations using analytical gradient techniques.[38] At each step in the optimization, the estimate of the true Hessian is improved by an updating procedure using the difference of current and previous gradients in relation to the difference in geometries. Depending on the type of calculation, different Hessian update schemes are used. For equilibrium geometry optimizations and intrinsic reaction coordinate (IRC) calculations with ADF, the Broyden–Fletcher–Goldfarb–Shanno (BFGS) scheme[39] is used. For transition state searches with QUILD, a weighted combination of Powell symmetric-Broyden (PSB)[40] and Murtangh–Sargent (Symmetric Rank-One, SR1)[41] is used, as developed by Bofill.[42] Energy minima have been verified through vibrational analysis.[43] All minima were found to have zero imaginary frequencies, whereas all transition states have one. The relative energies in the present study refer to the electronic energies without zero-point energy correction.

### Activation-strain analyses

Insight into the energetics is obtained through activation-strain analyses. The activation strain model[28] is a fragment-based approach to understand the bonding energy Δ*E* of two fragments during a chemical process and to explain it in terms of the original fragments. Within this approach, the potential energy surface Δ*E*(*ζ*) is decomposed along the reaction coordinate *ζ* (or just at one point *ζ*, for example at the transition state, TS, where Δ*E*^≠^=Δ*E*_*ζ*=TS_) into the strain energy Δ*E*_strain_(*ζ*) that is associated with the geometrical deformation of the individual fragments as the process takes place, plus the actual interaction energy Δ*E*_int_(*ζ*) between the deformed fragments [Eq. (3)].


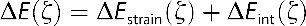
(3)

**Figure 1:**
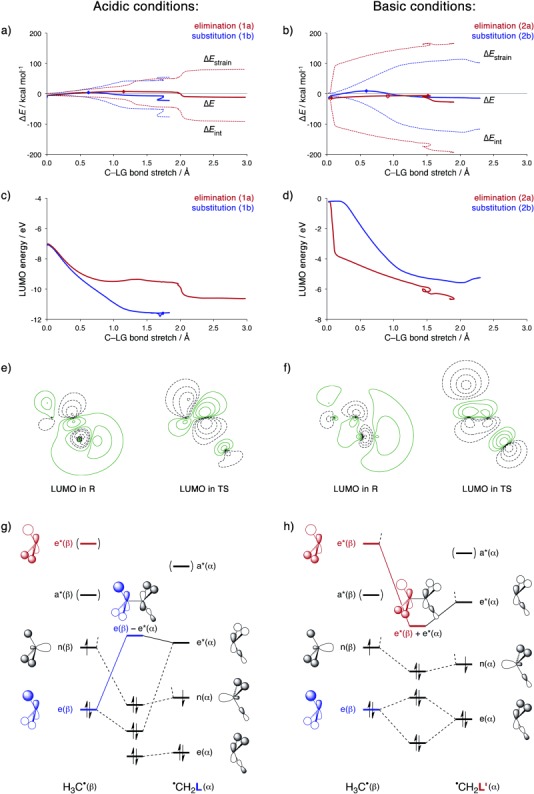
Activation-strain analyses of model reactions (a, b; dot designates transition state, TS), substrate-LUMO energies along reaction coordinate (c,d), substrate-LUMO contour plots (e,f) and schematic substrate-LUMO composition (g,h) under acidic and basic conditions, based on ZORA-OLYP/TZ2P computations (see text).

The interaction energy Δ*E*_int_(*ζ*) between the deformed fragments is further analyzed in the conceptual framework provided by the Kohn–Sham molecular orbital method.[44] We find that donor–acceptor interactions between occupied orbitals on one fragment with unoccupied orbitals on the other fragment, including the HOMO–LUMO interactions, play an important role in Δ*E*_int_ and in determining reactivity trends. The PyFrag program was used to facilitate the analyses along the potential energy surfaces.[45] Contour values used for the plots of the LUMOs in Figure 1 are ±0.02, ±0.05, ±0.1, ±0.2 and ±0.5.

## Results and Discussion

### Reactant complexes

**Scheme 2:**
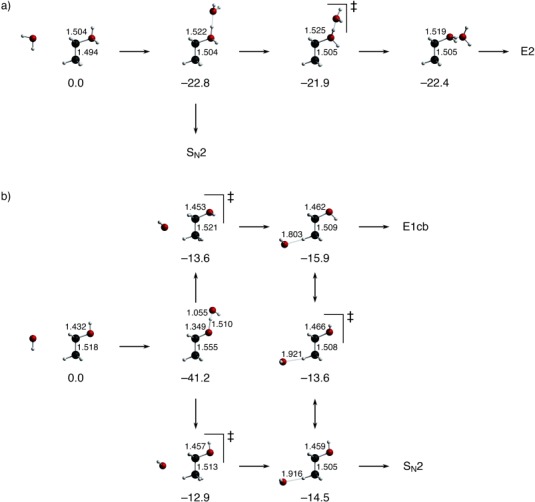
Relative energies (in kcal mol^−1^) and selected bond lengths (in Å) of isomeric reactant complexes for elimination and substitution reactions of a) H_2_O+C_2_H_5_OH_2_^+^, and b) OH^−^+C_2_H_5_OH, computed at ZORA-OLYP/TZ2P (see also Scheme 3).

Our ZORA-OLYP/TZ2P computations show that our model elimination and substitution reactions proceed via the formation of reactant complexes that, both under acidic and basic conditions, can rearrange into various variants. These complexes are shown in Scheme 2, which also provides relative energies and key geometry parameters of each stationary point (for full structural data and energies, see Table S1 in the Supporting Information). The most stable encounter complex in all four reactions features a hydrogen bond between H_2_O [Eqs. (1 a) and (1 b)] or OH^−^ [Eqs. (2 a) and (2 b)] and a hydrogen atom of the leaving group of the substrate. An obvious side reaction for our model systems R−OH_2_^+^ and R−OH is deprotonation of the leaving group, which is 22.0 kcal mol^−1^ endothermic in the former [Eq. (4 a)] and 21.7 kcal mol^−1^ *exothermic* in the latter case [Eq. (4 b)].[46]


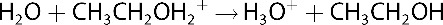
(4a)


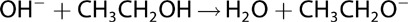
(4b)

Note however that this side reaction is not of interest in the present work, which serves to model the competition between elimination and substitution pathways of the more common and ubiquitous reactions of ethers R−OR′, amides R−NR′R“ and, in general, substrates R−L with a leaving group that is not easily deprotonated under the reaction conditions. In the following, we therefore focus on the elimination and substitution reaction pathways [Eqs. (1) and (2)].

In the acidic case [Eq. (1 a,b)], the most stable hydrogen-bonded reactant complex is bound by −22.8 kcal mol^−1^ and directly connected to the S_N_2 transition states (Scheme 2 a). On the other hand, along the E2 pathway, this reactant complex first undergoes a change in conformation via a low-barrier (0.9 kcal mol^−1^) rotation of the OH⋅⋅⋅H_2_O moiety around the C^α^−O bond to yield a slightly (0.4 kcal mol^−1^) less stable species. The latter again directly leads to the E2 transition state (Scheme 2 a). Under basic conditions [Eq. (2 a,b)], the lowest-energy reactant complex is even more stable, featuring a hydrogen-bond strength of −41.2 kcal mol^−1^. Prior to entering into the elimination and substitution channels, the base has to migrate first from the leaving group to a β hydrogen, yielding pre-reactive complexes at −15.9 and −14.5 kcal mol^−1^, respectively (Scheme 2 b). Essentially, these complexes differ only in the orientation of leaving group, and are easily interconverted via relatively low-barrier rearrangements.

### Reaction profiles

**Scheme 3:**
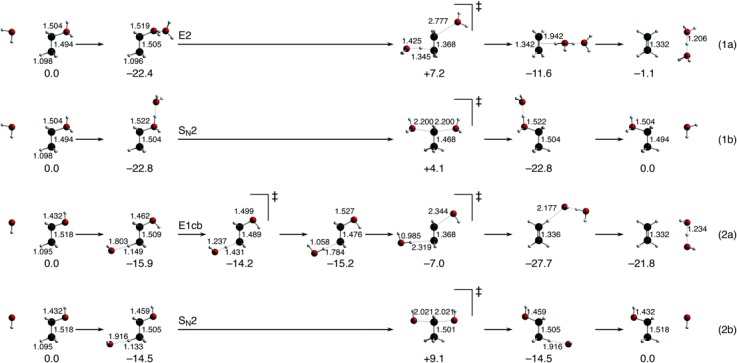
Relative energies (in kcal mol^−1^) and selected bond lengths (in Å) of stationary points along the lowest energy elimination and substitution pathways of H_2_O+C_2_H_5_OH_2_^+^ [Eq. (1 a,b)] and of OH^−^+C_2_H_5_OH [Eq. (2 a,b)], computed at ZORA-OLYP/TZ2P (see also Scheme 2).

The key stationary points and transition states (TS) for all four reaction paths, as they emerge from our ZORA-OLYP/TZ2P computations, are schematically displayed in Scheme 3, including relative energies and selected geometric data (for full structural and energy data, see Table S2 in the Supporting Information). Here we note that Scheme 3 shows the lowest-energy pathways. Alternative pathways exist in which stationary points (including the TS) adopt different conformations in which one or both of the OH or H_2_O groups are rotated. Typically these are only a few tenths of a kcal mol^−1^ higher in energy and will not be discussed here. For acidic conditions, we find that elimination [Eq. (1 a)] is outperformed by substitution [Eq. (1 b)], with reaction barriers of +7.2 versus +4.1 kcal mol^−1^, respectively. However, going to basic conditions, the reaction barrier for elimination [Eq. (2 a)] becomes −7.0 kcal mol^−1^ and thus drops below that for substitution [Eq. (2 b)], which assumes the significantly higher value of +9.1 kcal mol^−1^ (see Scheme 3). Thus, our model systems nicely reproduce the experimental observation that the preferred pathway often shifts from substitution to elimination if one goes from acidic to basic conditions. Before we provide detailed activation-strain analyses of this phenomenon, we will discuss in the following the mechanistic features and reaction profiles in more detail.

Under acidic conditions, we find that the elimination reaction proceeds in a concerted manner, via an E2 mechanism which is characterized by a double-well potential energy surface. One TS, at +7.2 kcal mol^−1^ relative to reactants, separates the aforementioned hydrogen-bonded reactant complex at −22.4 kcal mol^−1^ from the product complex at −11.6 kcal mol^−1^ (see Scheme 3). The product complex consists of a H_3_O^+^⋅⋅⋅H_2_O moiety interacting with ethylene. Dissociation yields the product olefin +H_3_O^+^⋅⋅⋅H_2_O at −1.1 kcal mol^−1^ relative to the reactants, which makes the overall elimination process slightly exothermic (see Scheme 3). The competing substitution also proceeds via a double-well potential energy surface, as is common for gas-phase S_N_2 substitution at carbon.[47, 48] The barrier for substitution is +4.1 kcal mol^−1^ relative to reactants, that is, approximately 3 kcal mol^−1^ lower than that for the elimination pathway (see Scheme 3). Overall, the symmetric S_N_2 reaction is thermoneutral.

Under basic conditions, we find again a double-well potential energy surface for the symmetric, thermoneutral S_N_2 reaction, with a TS at +9.1 kcal mol^−1^, that is, only slightly higher than for the corresponding substitution pathway in the acidic case. At variance, the TS of the competing alkaline elimination reaction drops significantly and, interestingly, the elimination reaction now proceeds via an E1cb mechanism. Thus, we arrive at a triple-well15a potential energy surface, along which a β-proton transfer from substrate to base occurs in a separate reaction step prior to the expulsion of the leaving group. The occurrence of an E1cb mechanism is in line with the strong basicity of the hydroxide anion and the poor leaving-group ability of the hydroxyl group.[49] The first TS, associated with β-proton abstraction, is at −14.2 kcal mol^−1^ relative to reactants, that is, less than 2 kcal mol^−1^ above the preceding reactant complex. The resulting intermediate, at −15.2 kcal mol^−1^ relative to reactants, is a O−H⋅⋅⋅C^−^ hydrogen-bonded complex between the newly formed water and the ^−^C^β^H_2_CH_2_OH carbanion. The second and overall TS, at −7.0 kcal mol^−1^ relative to reactants, leads to the expulsion of the leaving group. We note that the E1cb potential energy surface around the TS for leaving-group expulsion is extremely flat and may be considered a transition plateau, in line with earlier 2D explorations of potential energy surfaces of anionic E2 reactions.19a This causes the position of this TS along the reaction coordinate (but not its energy) to be sensitive for algorithmic differences (different Hessian update schemes, see computational details) between TS optimization and intrinsic reaction coordinate (IRC) exploration. Thus, the TS optimization yields a saddle point at a stretch in C^α^−LG bond of slightly less than 1 Å (open dot in PES for basic elimination in Figure 1 b), whereas the IRC scan reaches its highest point at a stretch of more than 1.5 Å (filled dot in PES for basic elimination in Figure 1 b). Importantly, the difference of less than 1 kcal mol^−1^ between the two energies (−7.0 and −6.1 kcal mol^−1^, respectively, relative to reactants) is significantly smaller than the effect that we are studying and therefore does not affect our conclusions. In the course of its expulsion, the leaving group forms a strongly hydrogen-bonded HO^−^⋅⋅⋅H_2_O moiety that interacts with ethylene, at −27.7 kcal mol^−1^ relative to reactants. Dissociation yields the product olefin+OH^−^⋅⋅⋅H_2_O at −21.8 kcal mol^−1^ relative to the reactants. This makes the overall elimination process under basic conditions rather exothermic.

In the following section, we use the activation-strain model to show how the barriers arise, how their relative order depends on nucleophilicity and leaving-group ability, and *why* the preferred reactivity shifts from substitution to elimination if one goes from acidic to basic conditions.

### Activation strain analyses: Acidic conditions

The ZORA-OLYP/TZ2P activation-strain diagrams (ASD) for all four model reactions are shown in Figure 1 a,b. Herein, the bold lines represent the reaction profile, that is, the relative energy profiles Δ*E*(*ζ*) as a function of the IRC projected onto the stretch of the bond between the α-carbon atom and the leaving group (C−LG). A dot on these lines indicates the TS. The relative energy is decomposed into a strain energy term, Δ*E*_strain_(*ζ*), which is the dashed line above Δ*E*=0, and the interaction energy Δ*E*_int_(*ζ*), represented by the dashed line below Δ*E*=0 (see [Eq. (3)] and Theoretical Methods for details).

Three general trends emerge from the ASDs of our model reactions: In the first place, the strain and the interaction curves show counteracting trends, if we go from acidic to basic conditions. Thus, for both elimination and substitution, the strain curve becomes more destabilizing and the interaction curve more stabilizing. This leads to a partial cancellation, and, as a result, changes in the total energy profile are comparatively small. Secondly, however, the changes in strain and interaction, if we go from acidic to basic conditions, are significantly larger for the elimination pathway. Thus, while in the acidic case the strain and interaction curves do not differ much for elimination and substitution, they adopt significantly larger absolute values for elimination under basic conditions. And third, the single most determining factor working in the direction of the overall shift in preferential reactivity from substitution in acidic to elimination in basic conditions is the enormous strengthening in base–substrate interaction in the latter case. In the following, we address the electronic mechanisms behind these trends.

The ASD for the acidic case shows that the barrier for the elimination reaction is not only higher than for the substitution reaction (+7.2 kcal mol^−1^ versus +4.1 kcal mol^−1^) but also occurs at a much later stage (see Figure 1 a). This is a consequence of the fact that, although the proton abstraction and leaving-group expulsion occur in a concerted manner, they do not occur synchronously: leaving-group expulsion is ahead of proton abstraction. One factor working in this direction is a relatively weakly pulling base, H_2_O (with a proton affinity of only 164.5 kcal mol^−1^, calculated from enthalpies at 298.15 K and 1 atm), and an excellent leaving group, also H_2_O, which makes for a rather soft C−LG bond. A deeper reason for the late, E1-like elimination mechanism is revealed when we inspect the Kohn–Sham orbital electronic structure of the substrate. For this purpose, we describe the molecular orbitals (MOs) of the CH_3_CH_2_L substrate (L=^+^OH_2_) in terms of a methyl and a substituted methyl radical, ^.^CH_3_(β)+^.^CH_2_L(α) (see Figure 1 g, α and β label the methyl fragment containing C^α^ and C^β^, respectively). Under acidic conditions, the substrate is protonated at the leaving group, and this stabilizes the corresponding ^.^CH_2_L(α) fragment molecular orbitals (FMOs) considerably.

This has an important consequence. The empty e*(α) FMO drops so much in energy that it begins to interact predominantly with the occupied e(β) instead of its direct counterpart e*(β), and the LUMO of the CH_3_CH_2_L substrate becomes mainly the antibonding e(β)−e*(α) combination (see Figure 1 g). Thus, the LUMO keeps its regular C^α^−L antibonding character stemming from e*(α),28c but it becomes C^β^−H *bonding* (instead of C^β^−H antibonding) due to e(β). This can also be nicely seen in the quantitative contour plots of the CH_3_CH_2_OH_2_^+^ LUMO in Figure 1 e, in particular, as one approaches the TS. Thus, the interaction with the base does not favor C^β^−H bond rupture, and β-proton abstraction occurs late when the C^α^−L stretch is advanced and quite some strain has already been built up. Furthermore, once the C^β^−H bond begins to stretch, this deformation has an energy-raising effect on the LUMO (because this orbital is C^β^−H bonding) and thus counteracts the energy-lowering effect of C^α^−L dissociation. This is at variance with the situation for the S_N_2 pathway along which the LUMO energy drops more significantly because only the C^α^−L has to break. The earlier and more pronounced decrease in LUMO energy along the S_N_2 pathway is plotted in Figure 1 c. All together, these effects favor an earlier onset of the base–substrate interaction along the S_N_2 than along the E2 pathway (see Figure 1 a). The result is the late, E1-like E2 elimination with a somewhat higher barrier than the corresponding S_N_2 substitution under acidic conditions.

### Activation strain analyses: Basic conditions

The ASD for basic conditions shows the aforementioned amplification of destabilizing strain and stabilizing interaction, especially for elimination, as compared with the ASD for acidic conditions (see Figure 1 b). The increase in the strain curve along the S_N_2 pathway is caused by the fact that the C^α^−OH bond in ethanol is significantly stronger (with a homolytic bond dissociation energy, BDE, of +93.7 kcal mol^−1^) than the C^α^−OH_2_^+^ bond (heterolytic BDE=+32.7 kcal mol^−1^). The stronger interaction results from the higher-energy HOMO of OH^−^, which makes this species a more strongly binding electron-donating agent than H_2_O in the interaction with an electron-accepting substrate.[29, 46] The increase in interaction would have been even higher if the substrate would not also change from protonated to non-protonated ethanol. The neutral substrate in the basic case has a higher-energy LUMO than the former in which all orbitals are lowered in energy due to the net positive potential. The two effects nearly cancel and, therefore, the S_N_2 reaction barrier of +9.1 kcal mol^−1^ under basic conditions is not very different from the S_N_2 barrier of +4.1 kcal mol^−1^ under acidic conditions.

Before continuing with the ASD for the elimination process, we first elaborate on the nature of the substrates LUMO. The latter undergoes a qualitative change in character if we go from the acidic to the basic situation, and this change plays a key role in understanding the shift in reactivity towards elimination. Again, we describe the MOs of the CH_3_CH_2_L’ substrate (this time, L′=OH) in terms of a methyl and a substituted methyl radical, ^.^CH_3_(β)+^.^CH_2_L′(α) (see Figure 1 h). Under basic conditions, the substrate is no longer protonated at the leaving group and, as a result, the corresponding ^.^CH_2_L′(α) FMOs are considerably higher in energy than in the acidic case. Thus, the e*(α) approaches e*(β), its counterpart on ^.^CH_3_(β), from below and the overall LUMO of the substrate CH_3_CH_2_L’ becomes mainly e*(β)+e*(α), that is, the bonding combination of the two empty methyl FMOs. Therefore, under basic conditions, the substrate LUMO is σ* antibonding in both the C^α^−LG and the C^β^−H bond (see Figure 1 h). This can also be clearly recognized in the quantitative contour plots of this LUMO as the substrate adopts the geometry that it has in the TS for elimination (see Figure 1 f).

Now, we return to the ASD. The combined C^α^−LG as well as C^β^−H antibonding character of the substrate LUMO leads to a completely different behavior along the elimination pathway under basic conditions. In this case, the interaction with the approaching base OH^−^ induces a weakening not only in the C^α^−LG bond but also in the C^β^−H bond, especially after this bond has been slightly extended (see “LUMO in TS” in Figure 1 f which also holds for slightly stretched C^β^−H distances). Furthermore, the stretching of the C^β^−H bond that goes with the elimination pathway now contributes to a lowering of the LUMO energy which therefore drops much faster than along the S_N_2 pathway, that is, exactly the other way around than under acidic conditions (cf. Figure 1 c,d). On one hand, the simultaneous stretching of C^β^−H and C^α^−LG that is now induced by the base–substrate interaction causes the strain to increase more pronouncedly. However, it also makes the interaction curve gain in stabilization significantly more steeply. Eventually, it is the better electron-donating capability of OH^−^ (with a proton affinity of 400.7 kcal mol^−1^, calculated from enthalpies at 298.15 K and 1 atm) in combination with the rapidly dropping LUMO energy along the elimination pathway that makes the base–substrate interaction pull the barrier of this pathway below that for S_N_2 substitution (cf. Figure 1 a,b).

Finally, the strong increase in interaction and, in particular, the C^β^−H antibonding character of the substrate LUMO also causes β-proton transfer to run ahead of leaving-group expulsion. This is so, even to the extent where β-proton transfer becomes a separate first step in the reaction mechanism, followed by leaving-group expulsion from the carbanion intermediate in a second step. Thus, our model reactions show a shift from an E1-like E2 elimination that is dominated by S_N_2 substitution under acidic conditions, to an E1cb elimination that dominates S_N_2 substitution under basic conditions.

## Conclusions

Our computations confirm the often observed shift from substitution to elimination when changing from acidic to more basic conditions. This follows from our theoretical analyses based on relativistic density functional theory (DFT) of the mutual competition between base-induced 1,2-elimination and nucleophilic substitution in model systems that represent, in a generic manner, one particular reaction under the two different conditions, namely, H_2_O+CH_3_CH_2_OH_2_^+^ for acidic and OH^−^+CH_3_CH_2_OH for basic conditions. In particular, we find that the elimination pathway in our model systems goes from an E1-like E2 mechanism that is dominated by S_N_2 substitution to an E1cb mechanism that prevails over S_N_2 substitution.

Our activation-strain analyses (ASA) reveal that the dominant cause for the above shift from nucleophilic (substitution) to protophilic reactivity (elimination) is an enormous gain in stabilizing interaction between the reactants if one goes to basic conditions, in particular in the case of elimination. The enhanced interaction is a direct consequence of the fact that the base (or nucleophile) changes from H_2_O to OH^−^. The latter has a significantly higher-energy HOMO and thus enters into more stabilizing interactions with a substrate LUMO. In the case of S_N_2 substitution, the enhanced interaction is approximately canceled by the more destabilizing strain that arises as we go from a relatively weak carbon−leaving-group bond under acidic (C^α^−OH_2_^+^) to a stronger one under basic conditions (C^α^−OH). Thus, the S_N_2 barrier changes comparatively little.

On the other hand, the enhancement of the interaction, from H_2_O to OH^−^, is significantly more pronounced for elimination and pulls the corresponding barrier below that for substitution. Interestingly, there is a fundamental reason why elimination benefits more from a higher-energy HOMO of the base under basic conditions, namely, the fact that also the substrate LUMO changes its character such that it favors the elimination pathway when it interacts with an attacking base. Thus, the LUMO goes from C^β^−H bonding and C^α^−LG antibonding under acidic conditions (i.e., in CH_3_CH_2_OH_2_^+^) to σ* antibonding in both bonds, C^β^−H and C^α^−LG, under basic conditions (i.e., in CH_3_CH_2_OH). Therefore, under acidic conditions, the HOMO–LUMO interaction between base and substrate does *not* assist C^β^−H rupture and the LUMO is *not* stabilized as the C^β^−H bond eventually breaks. At variance, under basic conditions, the HOMO–LUMO interaction between base and substrate *does* assist C^β^−H rupture and the LUMO *is* stabilized as the C^β^−H bond begins to stretch, right from the onset of the elimination process.

## References

[1a] Smith MB (2013). March's Advanced Organic Chemistry: Reactions, Mechanisms, and Structure.

[1b] Carey FA, Sundberg RJ (2007). Advanced Organic Chemistry, Part A.

[1c] Ingold C (1969). Structure and Mechanism in Organic Chemistry.

[2] DePuy CH, Gronert S, Mulin A, Bierbaum VM (1990). J. Am. Chem. Soc.

[3] Bickelhaupt FM, de Koning LJ, Nibbering NMM (1993). J. Org. Chem.

[4] Uggerud E, Bache-Andreassen L (1999). Chem. Eur. J.

[5a] Flores AE, Gronert S (1999). J. Am. Chem. Soc.

[5b] Gronert S, Pratt LM, Mogali S (2001). J. Am. Chem. Soc.

[5c] Gronert S (2003). Acc. Chem. Res.

[5d] Gronert S, Fagin AE, Okamoto K, Mogali S, Pratt LM (2004). J. Am. Chem. Soc.

[6] Villano SM, Kato S, Bierbaum VM (2006). J. Am. Chem. Soc.

[7] Ridge DP, Beauchamp JL (1974). J. Am. Chem. Soc.

[8] DePuy CH, Bierbaum VM (1981). J. Am. Chem. Soc.

[9] Van Doorn R, Jennings KR (1981). Org. Mass Spectrom.

[10] De Koning LJ, Nibbering NMM (1987). J. Am. Chem. Soc.

[11a] Lum RC, Grabowski JJ (1988). J. Am. Chem. Soc.

[11b] Lum RC, Grabowski JJ (1992). J. Am. Chem. Soc.

[12] Jones ME, Ellison GB (1989). J. Am. Chem. Soc.

[13] Gronert S (2001). Chem. Rev.

[14] Laerdahl JK, Uggerud E (2002). Int. J. Mass Spectrom.

[15a] Bickelhaupt FM, Buisman GJH, de Koning LJ, Nibbering NMM, Baerends EJ (1995). J. Am. Chem. Soc.

[15b] Bickelhaupt FM, de Koning LJ, Nibbering NMM (1993). Tetrahedron.

[16] Raghavachari K, Chandrasekhar J, Burnier RC (1984). J. Am. Chem. Soc.

[17] Occhiucci G, Speranza M, De Koning LJ, Nibbering NMM (1989). J. Am. Chem. Soc.

[18] Uggerud E (2003). Top. Curr. Chem.

[19a] Bickelhaupt FM, Baerends EJ, Nibbering NMM, Ziegler T (1993). J. Am. Chem. Soc.

[19b] Bickelhaupt FM, Baerends EJ, Nibbering NMM (1996). Chem. Eur. J.

[20a] Laerdahl JK, Uggerud E (2003). Org. Biomol. Chem.

[20b] Laerdahl JK, Bache-Andreassen L, Uggerud E (2003). Org. Biomol. Chem.

[21] Laerdahl JK, Civcir PU, Bache-Andreassen L, Uggerud E (2006). Org. Biomol. Chem.

[22] Uggerud E (2006). J. Phys. Org. Chem.

[23] Adlhart C, Uggerud E (2006). Phys. Chem. Chem. Phys.

[24] Uggerud E (2006). Chem. Eur. J.

[25] Civcir PU (2008). J. Mol. Struct.

[26] Uggerud E (1999). J. Chem. Soc. Perkin Trans. 2.

[27] Wu X-P, Sun X-M, Wei X-G, Ren Y, Wong N-B, Li W-K (2009). J. Chem. Theory Comput.

[28a] van Zeist >WJ, Bickelhaupt FM (2010). Org. Biomol. Chem.

[28b] de Jong GTh, Bickelhaupt FM (2007). J. Chem. Theory Comput.

[28c] Bickelhaupt FM (1999). J. Comput. Chem.

[29] Bento AP, Bickelhaupt FM (2008). J. Org. Chem.

[30a] te Velde G, Bickelhaupt FM, Baerends EJ, Fonseca Guerra C, van Gisbergen SJA, Snijders JG, Ziegler T (2001). J. Comput. Chem.

[30b] Fonseca Guerra C, Snijders JG, te Velde G, Baerends EJ (1998). Theor. Chem. Acc.

[30c] Baerends EJ, Ziegler T, Autschbach J, Bashford D, Bérces A, Bickelhaupt FM, Bo C, Boerrigter PM, Cavallo L, Chong DP, Deng L, Dickson RM, Ellis DE, van Faassen M, Fan L, Fischer TH, Fonseca Guerra C, Ghysels A, Giammona A, van Gisbergen SJA, Götz AW, Groeneveld JA, Gritsenko OV, Grüning M, Gusarov S, Harris FE, van den Hoek P, Jacob CR, Jacobsen H, Jensen L, Kaminski JW, van Kessel G, Kootstra F, Kovalenko A, Krykunov MV, van Lenthe E, McCormack DA, Michalak A, Mitoraj M, Neugebauer J, Nicu VP, Noodleman L, Osinga VP, Patchkovskii S, Philipsen PHT, Post D, Pye CC, Ravenek W, Rodríguez JI, Ros P, Schipper PRT, Schreckenbach G, Seldenthuis JS, Seth M, Snijders JG, Solà M, Swart M, Swerhone D, te Velde G, Vernooijs P, Versluis L, Visscher L, Visser O, Wang F, Wesolowski TA, van Wezenbeek EM, Wiesenekker G, Wolff SK, Woo TK, Yakovlev AL http://www.scm.com/.

[31a] Swart M, Bickelhaupt FM (2006). Computer software (QUILD).

[31b] Swart M, Bickelhaupt FM (2006). Int. J. Quantum Chem.

[31c] Swart M, Solà M, Bickelhaupt FM (2007). J. Comput. Chem.

[32a] Boerrigter PM, te Velde G, Baerends EJ (1988). Int. J. Quantum Chem.

[32b] te Velde G, Baerends EJ (1992). J. Comp. Phys.

[33] van Lenthe E, Baerends EJ (2003). J. Comput. Chem.

[34a] Handy NC, Cohen AJ (2001). Mol. Phys.

[34b] Handy NC, Cohen AJ (2002). J. Chem. Phys.

[35a] Lee C, Yang W, Parr RG (1988). Phys. Rev. B.

[35b] Johnson BG, Gill PMW, Pople JA (1993). J. Chem. Phys.

[35c] Russo TV, Martin RL, Hay PJ (1994). J. Chem. Phys.

[36a] van Lenthe E, Baerends EJ, Snijders JG (1994). J. Chem. Phys.

[36b] van Lenthe E, van Leeuwen R, Baerends EJ, Snijders JG (1996). Int. J. Quantum Chem.

[37] Bento AP, Solà M, Bickelhaupt FM (2008). J. Chem. Theory Comput.

[38] Versluis L, Ziegler T (1988). J. Chem. Phys.

[39a] Broyden CG (1970). J. Inst. Math. Its Appl.

[39b] Fletcher R (1970). Comput. J.

[39c] Goldfarb D (1970). Math. Comput.

[39d] Shanno DF (1970). Math. Comput.

[40a] Powell MJD, Rosen JB, Mangasarian OL, Ritter K (1970). in Nonlinear Programming.

[40b] Powell MJD (1971). Math. Prog.

[41] Murtagh B, Sargent RWH (1970). Comput. J.

[42] Bofill JM (1994). J. Comput. Chem.

[43a] Bérces A, Dickson RM, Fan L, Jacobsen H, Swerhone D, Ziegler T (1997). Comput. Phys. Commun.

[43b] Jacobsen H, Bérces A, Swerhone D, Ziegler T (1997). Comput. Phys. Commun.

[43c] Wolff SK (2005). Int. J. Quantum Chem.

[44a] Bickelhaupt FM, Baerends EJ, Lipkowitz KB, Boyd DB (2000). in Reviews in Computational Chemistry.

[44b] Bickelhaupt FM, Nibbering NMM, van Wezenbeek EM, Baerends EJ (1992). J. Phys. Chem.

[44c] Bickelhaupt FM, Diefenbach A, de Visser SP, de Koning LJ, Nibbering NMM (1998). J. Phys. Chem. A.

[44d] Ziegler T, Rauk A (1979). Inorg. Chem.

[45] van Zeist WJ, Fonseca Guerra C, Bickelhaupt FM (2008). J. Comput. Chem.

[46] Swart M, Bickelhaupt FM (2006). J. Chem. Theory Comput.

[46b] Swart M, Bickelhaupt FM (2006). J. Comput. Chem.

[46c] Swart M, Bickelhaupt FM (2007). Eur. J. Inorg. Chem.

[47] Olmstead WN, Brauman JI (1977). J. Am. Chem. Soc.

[48a] van Bochove MA, Bickelhaupt FM (2008). Eur. J. Org. Chem.

[48b] van Bochove MA, Swart M, Bickelhaupt FM (2006). J. Am. Chem. Soc.

[49] Saunders Jr WH (1976). Acc. Chem. Res.

