# Supplementary material for: Understanding E2 versus SN2 Competition under Acidic and Basic Conditions
Source: ChemistryOpen. 2014 Jan 29;3(1):29–36. doi: 10.1002/open.201300043 (PMC3943610; doi:10.1002/open.201300043)
Supplement: Supplementary file 1 [file open0003-0029-sd1.pdf]

ChemistryOPEN

Supporting Information

© 2014 The Authors. Published by Wiley-VCH Verlag GmbH & Co. KGaA, Weinheim

Understanding E2 versus S_N2 Competition under Acidic and Basic Conditions

Lando P. Wolters,[a] Yi Ren,*[b] and F. Matthias Bickelhaupt*[a, c]

open_201300043_sm_miscellaneous_information.pdf

Contents

Table S1. Cartesian coordinates and ADF total energies of stationary points shown in Scheme 2 of the main text, computed at ZORA-OLYP/TZ2P.

Table S2. Cartesian coordinates and ADF total energies of stationary points shown in Scheme 3 of the main text, computed at ZORA-OLYP/TZ2P.

Table S1. Cartesian coordinates (in Å) and ADF total energies (in kcal/mol) of stationary points shown in Scheme 2 of the main text, computed at ZORA-OLYP/TZ2P.

H^+			[+291.40]
H	0.00000000	0.00000000	0.00000000

OH'			[-206.67]
H	0.00000000	0.00000000	0.30539878
O	0.00000000	0.00000000	1.27426264

H_2O			[-322.63]
O	0.00000000	0.00000000	0.34827273
H	0.00000000	0.75975973	-0.24744459
H	0.00000000	-0.75975973	-0.24744459

C_2H_5OH			[-1047.39]
O	-1.44346648	0.49400637	0.00000000
C	-0.33906458	-0.41827582	0.00000000
H	-0.37626512	-1.06824852	0.88919587
H	-0.37626512	-1.06824852	-0.88919587
C	0.94912136	0.38423920	0.00000000
H	1.81258308	-0.28944182	0.00000000
H	1.00781841	1.02253012	-0.88739487
H	1.00781841	1.02253012	0.88739487
H	-2.25581628	-0.02655572	0.00000000

$C_2H_5OH_2^+$			[-949.10]
O	-1.37445459	0.31030934	-0.11275744
C	-0.09466187	-0.60488296	0.01930971
H	-0.23459199	-1.14391898	0.95868090
H	-0.22130076	-1.25597908	-0.84679883
C	1.11030399	0.27732633	-0.03343114
H	1.98147849	-0.38898138	0.01458188
H	1.17111625	0.83939689	-0.96873978
H	1.17656102	0.95100798	0.82738519
H	-2.20718555	-0.19479282	-0.02605183
H	-1.37749583	1.02508282	0.55510335

acidic, first RC			[-1294.49]
O	-0.56917920	-0.38584562	0.42321123
C	-0.36903507	0.56718129	-0.74612144
O	0.26098362	0.49272120	2.60346205
H	-0.91825830	1.45314208	-0.42462467
C	-0.92099897	-0.05901985	-1.99675468
H	0.70230536	0.77776860	-0.79613940
H	-0.35139766	0.45419550	3.35314209
H	-0.23415561	-1.27355632	0.20798824
H	-0.79612712	0.66232165	-2.81171120
H	-1.98658430	-0.28380427	-1.90262434
H	-0.37295542	-0.96444919	-2.28040241
H	1.14547436	0.32321753	2.95906013
H	-0.16913307	-0.04970333	1.34574311

acidic, TS			[-1293.60]
C	-0.67804583	-0.28266756	-0.89416135
C	-1.25453710	1.04756189	-1.29664011
H	-0.53110267	1.86278543	-1.19397890
H	-2.16856760	1.28186489	-0.74029686
H	0.25868746	-0.52381041	-1.39973380
H	-1.38395472	-1.10845660	-0.98870813
O	-0.32676733	-0.29111081	0.59008420
H	-1.53052322	0.98372764	-2.35504737
O	1.98836778	-1.04516256	1.10385085
H	-0.57358283	0.54810132	1.01321614
H	2.73240888	-0.44356093	1.25253681
H	0.67329847	-0.55553425	0.82367742
H	2.07864144	-1.76278415	1.74842836

acidic, second RC			[-1294.09]
C	-0.93279952	-0.35350478	-0.81083971
C	-0.78176048	1.08118118	-1.24083770
H	0.26015231	1.41385985	-1.23065109
H	-1.39406841	1.75980653	-0.63725588
H	-0.30508178	-1.05164084	-1.36636768
H	-1.96499708	-0.70738385	-0.81608806
O	-0.49369349	-0.58607567	0.62486682
H	-1.14256561	1.15422511	-2.27343827
O	1.97024930	-0.73631630	0.95269335
H	-0.93180762	0.03586100	1.23095037
H	2.52798917	0.00378158	1.23328093
H	0.55653038	-0.58438452	0.78401989
H	2.28995870	-1.51889683	1.42598613

basic, TS top left			[-1267.61]
O	-0.24843173	0.12884888	-0.09964213
C	0.21331560	3.55351613	0.39004402
O	-0.02092738	4.87823896	-0.16014893
H	-0.46974489	2.80401276	-0.03463588
C	1.63550215	3.04650371	0.20694743
H	-0.02252872	3.65472247	1.45624623
H	0.01790196	4.76952674	-1.11904054
H	-0.61924965	0.15530129	0.79342410
H	1.92083130	3.08640490	-0.85364400
H	1.64693046	1.98494749	0.49180466
H	2.36616852	3.63160367	0.78352453

basic, RC middle left			[-1217.15]
C	-0.70243342	0.69431407	0.97393926
C	0.34342280	0.84934302	-0.16587962
H	1.09708752	0.02158784	0.01733622
H	0.91682067	1.79015533	0.07809300
H	-1.42623946	1.52073628	0.93850988
H	-0.22698654	0.68265673	1.97174656
H	-1.26338143	-0.24163721	0.84332795
O	0.79412678	2.41527181	-3.15751538
O	-0.19515616	0.83623931	-1.40276546
H	0.42395930	1.75395417	-2.42306495
H	0.03480544	2.99414685	-3.29167436

basic, TS bottom left			[-1266.95]
O	-0.30099155	0.10998489	-0.16443643
C	0.18454322	3.53947803	0.26257565
O	0.12977407	4.92767175	-0.17530113
H	-0.43002374	2.88475168	-0.36903938
C	1.60783380	3.02755978	0.23037920
H	-0.21434902	3.46906046	1.28811986
H	-0.78928252	5.09105025	-0.41893687
H	-0.67418905	0.14052273	0.72761044
H	2.04726601	3.18857886	-0.76189142
H	1.55742915	1.94380048	0.40812091
H	2.24408262	3.52414509	0.97647518

basic, RC top right			[-1269.91]
O	-2.23227871	0.09625108	-0.03010958
C	-0.77870145	0.03974890	0.11189568
O	2.86242457	0.15901607	-0.44540088
H	-0.51321175	0.89918235	0.73932127
C	0.00685558	0.07295693	-1.17648631
H	-0.52244879	-0.86834764	0.68174348
H	2.91653679	-0.64633494	0.08952383
H	-2.41021664	-0.29291877	-0.89587333
H	-0.23666584	0.97286965	-1.76092739
H	-0.21383132	-0.81443096	-1.79348583
H	1.13073877	0.08029273	-0.93971898

basic, TS middle right			[-1267.65]
O	-0.02534482	0.00906068	0.00535967
C	0.02615766	3.68433997	-0.01289011
O	0.35363844	5.11287063	0.00987013
H	-0.48427989	3.47838413	-0.96384540
C	1.22639773	2.78123257	0.12341660
H	-0.69565855	3.46461993	0.78678319
H	-0.16823075	5.50615881	0.71915354
H	-0.23280534	-0.09843290	0.94486080
H	1.94226398	2.96223604	-0.69078873
H	0.86753059	1.70807786	0.08186245
H	1.74213598	2.95762684	1.07876427

basic, RC bottom right			[-1268.52]
O	-0.00034054	0.00013977	0.00001147
C	-0.00018606	3.69181455	-0.00042805
O	0.38087604	5.09991205	0.00022838
H	-0.53833921	3.45673466	-0.93201298
C	1.19916587	2.79231526	0.12751291
H	-0.69639051	3.50349615	0.83315662
H	-0.43787890	5.60701409	-0.09936824
H	-0.35073857	-0.03370386	0.90191244
H	1.89778994	2.95824308	-0.70432712
H	0.84313585	1.71712677	0.10564970
H	1.73244281	2.98566196	1.06905113

Table S2. Cartesian coordinates (in Å) and ADF total energies (in kcal/mol) of stationary points shown in Scheme 3 of the main text, computed at ZORA-OLYP/TZ2P.

H^+ [+291.40]

H	0.00000000	0.00000000	0.00000000

$OH^•$ [-206.67]

H	0.00000000	0.00000000	0.30539878
O	0.00000000	0.00000000	1.27426264

H_2O [-322.63]

O	0.00000000	0.00000000	0.34827273
H	0.00000000	0.75975973	-0.24744459
H	0.00000000	-0.75975973	-0.24744459

H_3O^+ [-202.36]

O	0.00000000	0.00000000	0.26083848
H	-0.47030231	-0.81458750	-0.02480379
H	-0.47030231	0.81458750	-0.02480379
H	0.94060463	0.00000000	-0.02480379

$C_2H_5OH_2^+$ [-949.10]

O	-1.37445459	0.31030934	-0.11275744
C	-0.09466187	-0.60488296	0.01930971
H	-0.23459199	-1.14391898	0.95868090
H	-0.22130076	-1.25597908	-0.84679883
C	1.11030399	0.27732633	-0.03343114
H	1.98147849	-0.38898138	0.01458188
H	1.17111625	0.83939689	-0.96873978
H	1.17656102	0.95100798	0.82738519
H	-2.20718555	-0.19479282	-0.02605183
H	-1.37749583	1.02508282	0.55510335

C_2H_5OH [-1047.39]

O	-1.44346648	0.49400637	0.00000000
C	-0.33906458	-0.41827582	0.00000000
H	-0.37626512	-1.06824852	0.88919587
H	-0.37626512	-1.06824852	-0.88919587
C	0.94912136	0.38423920	0.00000000
H	1.81258308	-0.28944182	0.00000000
H	1.00781841	1.02253012	-0.88739487
H	1.00781841	1.02253012	0.88739487
H	-2.25581628	-0.02655572	0.00000000

C_2H_4 [-714.76]

C	0.66607542	0.00000000	0.00000000
C	-0.66607542	0.00000000	0.00000000
H	1.23774245	-0.92571703	0.00000000
H	1.23774245	0.92571703	0.00000000
H	-1.23774245	-0.92571703	0.00000000
H	-1.23774245	0.92571703	0.00000000

$H_2O–H_3O^+$ [-558.08]

O	-0.09749256	0.00210756	0.41148561
H	-0.62635773	-0.81049424	0.47082613
H	-0.68723286	0.76783118	0.51451046
H	0.71476134	0.07644291	-0.47677259
O	1.58124803	0.25037643	-1.29729602
H	1.45366988	-0.14212859	-2.17661608
H	2.48960681	0.06964367	-1.00240765

$H_2O–OH^•$ [-561.13]

O	-0.00831801	0.00942832	0.97832569
H	0.08333051	-0.91420707	1.79171535
H	0.65669808	0.62415005	1.31237809
H	0.84908904	-2.39595904	2.16987463
O	0.19248191	-1.83711661	2.60377392

Reaction 1a: RC [-1294.09]

C	-0.93279952	-0.35350478	-0.81083971
C	-0.78176048	1.08118118	-1.24083770
H	0.26015231	1.41385985	-1.23065109
H	-1.39406841	1.75980653	-0.63725588
H	-0.30508178	-1.05164084	-1.36636768
H	-1.96499708	-0.70738385	-0.81608806
O	-0.49369349	-0.58607567	0.62486682
H	-1.14256561	1.15422511	-2.27343827
O	1.97024930	-0.73631630	0.95269335
H	-0.93180762	0.03586100	1.23095037
H	2.52798917	0.00378158	1.23328093
H	0.55653038	-0.58438452	0.78401989
H	2.28995870	-1.51889683	1.42598613

Reaction 1a: TS [-1264.51]

C	-0.17457568	-0.33975096	0.03788756
C	0.43356883	0.61788999	-0.72644923
H	0.59323547	1.61996268	-0.32999907
H	0.51477539	0.49303281	-1.80558388
H	-0.33705066	-0.19576541	1.10301996
H	-0.41372066	-1.31860132	-0.37118471
O	-2.91702263	-0.35122443	0.47560849
H	1.50441937	-0.05397208	-0.26642020
O	2.80846820	-0.53955794	0.03970184
H	-3.49940763	-1.09422485	0.68377473
H	3.37701384	0.07042243	0.53751475
H	-3.51851499	0.33024904	0.14640969
H	3.33334998	-0.84328601	-0.71880615

Reaction 1a: PC [-1283.37]

C	0.52143867	1.68916219	-0.98962029
C	0.96143308	0.69712209	-1.77859714
H	2.02467091	0.50229441	-1.90947485
H	0.27656788	0.09520822	-2.37347128
H	1.20874748	2.33084490	-0.44158771
H	-0.53818833	1.92505718	-0.90819151
O	-1.60387629	-1.75329953	1.13777491
H	0.64834531	-0.04584782	0.10878786
O	0.59220081	-0.64759877	0.93407141
H	-1.79107506	-2.63950997	0.79500319
H	1.37032952	-1.22765493	0.97751090
H	-2.05678560	-1.67749350	1.99101307
H	-0.33600337	-1.18748845	1.01211347

Reaction 2a: RC [-1269.91]

O	-2.23227871	0.09625108	-0.03010958
C	-0.77870145	0.03974890	0.11189568
O	2.86242457	0.15901607	-0.44540088
H	-0.51321175	0.89918235	0.73932127
C	0.00685558	0.07295693	-1.17648631
H	-0.52244879	-0.86834764	0.68174348
H	2.91653679	-0.64633494	0.08952383
H	-2.41021664	-0.29291877	-0.89587333
H	-0.23666584	0.97286965	-1.76092739
H	-0.21383132	-0.81443096	-1.79348583
H	1.13073877	0.08029273	-0.93971898

Reaction 2a: TS deprotonation [-1268.23]

O	-2.22778591	0.07462133	-0.05406464
C	-0.74041393	0.05904852	0.13119855
O	2.71078958	0.12671934	-0.53483367
H	-0.51983320	0.93416726	0.76263990
C	0.10380220	0.06785327	-1.09557181
H	-0.54449284	-0.84066713	0.73726781
H	2.80936077	-0.58996316	0.10716620
H	-2.30714187	0.12176506	-1.01585661
H	-0.06086577	0.97682837	-1.69943507
H	-0.07227682	-0.82323401	-1.72215727
H	1.49782757	0.08481603	-0.77264913

Reaction 2a: Intermediate [-1269.17]

C	-1.12182104	0.02292484	-0.15140523
C	-0.15916133	1.10937938	0.11533877
H	-0.18356698	1.46481151	1.15809783
H	-0.25402260	1.95480922	-0.58510316
H	-0.96879412	-0.83070895	0.52866825
H	-1.03553472	-0.35074681	-1.18443389
H	2.49585563	-0.59574107	-0.37414595
H	1.54293030	0.61995336	-0.10313925
O	2.56011148	0.35659091	-0.22481059
O	-2.61022225	0.33195891	-0.00625252
H	-2.57426518	1.26326590	0.25226561

Reaction 2a: TS LG expulsion [-1261.05]

C	-0.81317377	0.28605319	-0.09233116
C	-0.16303743	1.45285297	0.20317674
H	0.03748257	1.74482202	1.23485299
H	-0.03827392	2.23758097	-0.54411407
H	-0.94059939	-0.49114053	0.65066912
H	-1.01582143	-0.00168509	-1.11627401
H	2.61781856	-0.74342867	-0.52273610
H	1.94859780	0.55503196	-0.13440237
O	2.84126964	0.16394504	-0.28017249
O	-3.14229496	0.02380139	-0.06572106
H	-3.23329622	0.94988108	0.19739739

Reaction 2a: PC [-1281.77]

C	-1.05375375	1.98229643	1.27023238
C	-0.28753707	3.07016508	1.15485532
H	-0.40355305	3.94254508	1.80065716
H	0.49772161	3.12950393	0.40067687
H	-1.83274785	1.95075406	2.03574432
H	-0.95534168	1.09402457	0.62448182
H	0.16546140	-1.32884950	-1.13964528
H	1.69067005	-1.03440385	-1.51716914
O	1.07286054	-1.77310411	-1.56877028
O	-0.99402489	-0.71899352	-0.58078181
H	-1.46813150	-0.42536300	-1.36924883

Reaction 1b: RC & PC [-1294.49]

O	-0.56917920	-0.38584562	0.42321123
C	-0.36903507	0.56718129	-0.74612144
O	0.26098362	0.49272120	2.60346205
H	-0.91825830	1.45314208	-0.42462467
C	-0.92099897	-0.05901985	-1.99675468
H	0.70230536	0.77776860	-0.79613940
H	-0.35139766	0.45419550	3.35314209
H	-0.23415561	-1.27355632	0.20798824
H	-0.79612712	0.66232165	-2.81171120
H	-1.98658430	-0.28380427	-1.90262434
H	-0.37295542	-0.96444919	-2.28040241
H	1.14547436	0.32321753	2.95906013
H	-0.16913307	-0.04970333	1.34574311

Reaction 1b: TS [-1267.66]

O	-2.11387904	0.00647288	0.25215714
C	0.02599211	0.08832290	-0.25202200
O	2.20431982	0.36245650	-0.10507476
H	-0.01995680	1.08031217	0.18009988
C	-0.08072254	-0.07489138	-1.70717689
H	0.15085715	-0.74825434	0.42214429
H	2.52145774	0.58894223	0.78411512
H	-2.51737103	-0.85996727	0.08116027
H	0.70015341	0.52543123	-2.19192239
H	-1.01893921	0.38090602	-2.04948569
H	-0.02114285	-1.11199813	-2.03850685
H	2.70971033	-0.42843156	-0.35370669
H	-2.31390236	0.19257780	1.18376924

Reaction 2b: RC & PC [-1268.52]

O	-0.00034054	0.00013977	0.00001147
C	-0.00018606	3.69181455	-0.00042805
O	0.38087604	5.09991205	0.00022838
H	-0.53833921	3.45673466	-0.93201298
C	1.19916587	2.79231526	0.12751291
H	-0.69639051	3.50349615	0.83315662
H	-0.43787890	5.60701409	-0.09936824
H	-0.35073857	-0.03370386	0.90191244
H	1.89778994	2.95824308	-0.70432712
H	0.84313585	1.71712677	0.10564970
H	1.73244281	2.98566196	1.06905113

Reaction 2b: TS [-1244.96]

O	-1.94529154	0.06417485	0.19837450
C	0.04549766	0.14510668	-0.13406784
O	2.06450600	0.22666325	-0.17446707
H	0.04045848	1.14867092	0.25732404
C	-0.07591559	-0.06073960	-1.61571196
H	0.14155319	-0.69602787	0.53143163
H	2.23200754	0.23937447	0.77848244
H	-1.93933054	0.06143410	1.16606673
H	0.72324081	0.48126280	-2.13245003
H	-1.05194161	0.29260991	-1.96391639
H	0.01670486	-1.12177789	-1.87157224
